# High-Affinity Target Binding Engineered via Fusion of a Single-Domain Antibody Fragment with a Ligand-Tailored SH3 Domain

**DOI:** 10.1371/journal.pone.0040331

**Published:** 2012-07-05

**Authors:** Annika Järviluoma, Tomas Strandin, Sebastian Lülf, Jérôme Bouchet, Anna R. Mäkelä, Matthias Geyer, Serge Benichou, Kalle Saksela

**Affiliations:** 1 Department of Virology, Haartman Institute, University of Helsinki, Helsinki, Finland; 2 Department of Physical Biochemistry, Max Planck Institute of Molecular Physiology, Dortmund, Germany; 3 Institut Cochin, CNRS (UMR8104), Université Paris Descartes, Paris, France; 4 Inserm U1016, Paris, France; 5 NEXT Biomed Technologies NBT Oy, Helsinki, Finland; 6 HUSLAB, Helsinki University Central Hospital, Helsinki, Finland; Technical University of Braunschweig, Germany

## Abstract

Monoclonal and recombinant antibodies are ubiquitous tools in diagnostics, therapeutics, and biotechnology. However, their biochemical properties lack optimal robustness, their bacterial production is not easy, and possibilities to create multifunctional fusion proteins based on them are limited. Moreover, the binding affinities of antibodies towards their antigens are suboptimal for many applications where they are commonly used. To address these issues we have made use of the concept of creating high binding affinity based on multivalent target recognition via exploiting some of the best features of immunoglobulins (Ig) and non-Ig-derived ligand-binding domains. We have constructed a small protein, named Neffin, comprised of a 118 aa llama Ig heavy chain variable domain fragment (VHH) fused to a ligand-tailored 57 aa SH3 domain. Neffin could be readily produced in large amounts (>18 mg/L) in the cytoplasm of *E. coli,* and bound with a subpicomolar affinity (*K_d_* 0.54 pM) to its target, the HIV-1 Nef protein. When expressed in human cells Neffin could potently inhibit Nef function. Similar VHH-SH3 fusion proteins could be targeted against many other proteins of interest and could have widespread use in diverse medical and biotechnology applications where biochemical robustness and strong binding affinity are required.

## Introduction

Specific recognition and strong binding to chosen target molecules is the cornerstone of modern therapeutic and diagnostic practices. Monoclonal antibody technology pioneered by Köhler and Milstein in the 1970’s revolutionized medical and other fields of immunodiagnostic development [1], and currently accounts for a significant portion of new drugs approved for treatment of major human diseases, such as cancer and autoimmune disorders [2], [3].

Subsequent progress in molecular biology has made it possible to generate recombinant antibodies with rationally altered binding properties and multifunctional fusion partners [4], [5]. Recombinant antibodies containing only the Fab fragment and single-chain antibodies (scFv) comprised only of the variable domains of heavy and light chains joined by a flexible linker peptide represent simpler and smaller alternatives to complete immunoglobulins. Fab and scFv proteins can be easily manipulated and often produced in relatively large amounts in prokaryotic expression systems. The possibility to select recombinant antibodies from synthetic libraries and to optimize their properties by random and targeted mutagenesis combined with powerful *in vitro* affinity selection schemes have been fruitfully exploited in various biotechnology applications. These approaches enable rational targeting of antibody binding, including target epitopes that might be poorly immunogenic, as well as overcoming the affinity ceiling of monoclonal antibodies. While most natural antibodies have *K_d_* values in the range of 10^−8^ to 10^−11^ M [6], [7], orders of magnitude tighter binding has been reported for optimized recombinant antibodies [8].

Despite these advantages, problems and limitations related to recombinant antibodies exist, which have hindered their widespread use. Due to the complex structure recombinant antibodies show challenging biophysical properties, and are lacking the robustness of ideal recombinant protein reagents [9], [10], [11], [12]. Accordingly, recombinant antibodies have poor stability under reducing conditions, such as the intracellular environment. Moreover, their antigen recognition can be sensitive for context-specific steric effects, thus limiting the freedom to create multifunctional fusion protein derivatives.

Therefore, several investigators have considered the use of non-Ig proteins as sources (“scaffold proteins”) for novel high affinity ligand binders via applying the same principles of sequence diversification and affinity selection successfully applied in recombinant antibody engineering. A growing number of proteins and protein domains, with normal functions either related or unrelated to protein interactions, have been established as suitable backbones for engineering of artificial proteins with useful binding specificities (for reviews, see [13], [14], [15]). Among the best validated examples of these are affibodies based on the Z-domain of staphylococcal protein A [16], monobodies based on the 10th extracellular domain of human fibronectin III [17], and DARPins (designed ankyrin repeat domains) comprised of an optimized target binding interface built from four to six ankyrin repeat modules with engineered binding properties [18].

Another attractive non-Ig scaffold is the SH3 domain [19], [20], representing a small (55–60 aa) protein module with a compact beta-sandwich fold lacking disulfide bridges, which can be easily expressed in large amounts and in soluble form in *E. coli.* By randomizing the non-conserved flexible loops of SH3 domains they have been successfully targeted for binding to diverse ligand proteins with low nanomolar affinities [21], [22], [23].

An alternative approach to address the challenges related to the biochemical properties of recombinant antibodies has been to exploit the ability of certain immunoglobulin variable domains to bind target antigens as independent monomeric units [24]. In particular, camelids and sharks naturally produce a class of antibodies comprised only of the heavy chain [25]. Variable domain fragments of camelid antibodies, termed VHH domains, nanobodies, or single-domain antibodies (sdAb), can bind to their cognate antigens with affinities comparable to regular antibodies, but due to their simpler architecture have advantageous biophysical properties (solubility, stability) [26], [27], [28], and offer attractive opportunities for further molecular design [29]. Remarkably, the typical length of an sdAb is only 120 amino acid residues, thus representing the most minimalistic form of an antibody.

Enhanced affinity in natural protein interactions is often achieved via combined use of multiple binding domains. Neri and colleagues have successfully exploited this principle in antibody engineering by creating heterodimeric proteins (dubbed CRAb for chelating recombinant antibody) built of two linker-connected scFv’s binding to adjacent non-overlapping epitopes in a common target antigen [30]. An impressive strength of binding (*K_d_* in low picomolar range) was obtained as a result of more than a 100-fold increase in affinity compared to either one of its scFv components.

However, it is evident that the problems related to antibody structure and biochemistry will increase rather than decrease upon fusing two scFv molecules together. Therefore, it would be an attractive idea to use heterologous (*i.e.* non-Ig-derived) ligand binding proteins as co-operating components of multi-domain constructs designed for high affinity target recognition. Indeed, increase in affinity and specificity by the formation of multivalent interactions is a well-known concept in modular protein interactions (see [31], [32]). Examples of this approach in protein engineering are the “affinity clamp” proteins constructed by Koide and colleagues based on optimized fibronectin domains fused with PDZ domains [33], and the “avimers” construted by Silverman and colleagues based on multimers of cell-surface receptor-derived A-domains [34].

In the present study we have created a promising new multi-domain protein with strongly cooperative target binding properties by combining some of the best concepts in antibody engineering and in non-Ig scaffold design. We have generated a fusion protein comprised of an sdAb fragment derived from a llama immunized against the HIV-1 pathogenicity factor Nef with a synthetic library-derived SH3 domain optimized for binding to Nef. The resulting small (<200 residues) polypeptide showed greatly enhanced binding to Nef compared to either one of its individual components alone, resulting in a subpicomolar binding affinity. This fusion protein, designated as ‘Neffin’, showed favorable biochemical and functional properties, could be easily produced in high amounts in *E. coli*, and acted as a potent intracellular inhibitor of Nef function in human cells.

## Results

### Construction of Neffin

Bivalent target recognition is an attractive concept for generating high affinity binding polypeptides for therapeutic and diagnostic applications. To exploit this strategy but to avoid problems related to poor expression, stability and solubility we chose to create a chimeric polypeptide comprised of a minimalistic antigen-binding Ig fragment fused to a small non-Ig protein-binding domain. To this end we combined a 118 amino acid llama-derived single domain Ig heavy chain variable domain fragment (VHH) with a 57 amino acid SH3 domain derived from the human Hck tyrosine kinase.

The VHH domain (termed sdAb19) was cloned from a llama immunized against the HIV-1 pathogenicity factor Nef, and has shown to be able to inhibit intracellular functions of Nef [35]. Likewise, the SH3 domain (SH3-B6) used here has been optimized for binding to HIV-1 Nef by manipulation of the amino acid sequence in the specificity-determining RT-loop region of Hck SH3 [22], and has been shown to be able to inhibit Nef as such [36], or as an improved fusion protein including a Nef-binding fragment from human CD4 [37]. We dubbed the resulting anti-Nef VHH-SH3 domain chimera Neffin.

**Figure 1:**
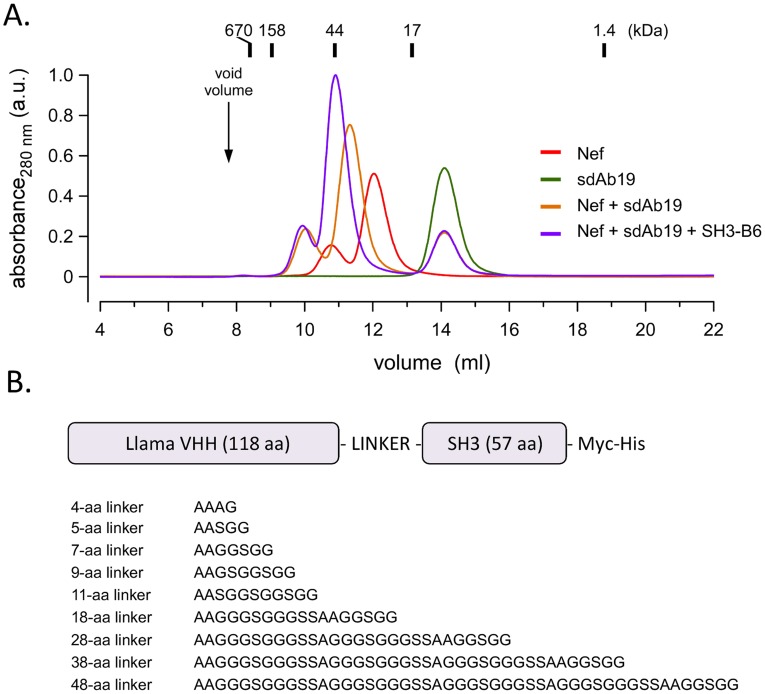
Design of a VHH-SH3 fusion protein (Neffin) targeted against HIV-1 Nef. (**A**) Size exclusion chromatography analysis confirming the expected molecular sizes for the monomeric Nef, sdAb19, and SH3-B6 proteins and for the dimeric sdAb19/Nef and the trimeric sdAb19/Nef/SH3-B6 complexes. (**B**) Domain organization of Neffin and the amino acid sequences of the different linkers tested for joining of sdAb19 and SH3-B6.

Since the exact target site in Nef is only known for SH3-B6 [38], but not for sdAb19, we first analyzed SH3 and sdAb19 binding to Nef by size-exclusion chromatography to ask if both protein domains could bind simultaneously to the surface of Nef. These experiments were performed using the core domain of Nef comprising residues 41–206 (Fig. 1A). Both Nef and sdAb19 run at elution volumes of their expected sizes indicating a monomeric dispersion of the two proteins. Addition of sdAb19 to Nef led to a marked increase in protein size displayed by an earlier elution volume, which corresponded to the tight complex formation between these proteins. Addition of SH3-B6 to Nef and sdAb19 furthermore increased the size of the protein eluate to an apparent size of about 44 kDa. This elution profile indicated the tripartite formation of the Nef–sdAb19–SH3-B6 complex (Fig. 1A). These results confirmed that binding of the SH3 domain to the PxxPxR motif in Nef and binding of sdAb19 to Nef is complementary and not mutually exclusive.

Having confirmed that SH3-B6 could bind to Nef simultaneously with sdAb19 we next tested a panel of Gly-Ser-containing linkers of different lengths introduced between SH3-B6 and sdAb19 to generate a fusion protein that would enable synergistic binding to Nef. Nine different Neffin constructs were generated in which sdAb19 was connected to SH3-B6 via linkers ranging from 4 to 48 aa in length (see Fig. 1B). Due to the modular nature of the SH3 fold it is relatively insensitive to the context where it is placed, and tolerates well fusion of heterologous sequences both at its N- and C-termini. Instead, the antigen binding capacity of single-domain antibody fragments might be compromised by foreign material appended to the N-terminus. Therefore, in all cases the Neffins were designed such that the sdAb19 was located N-terminally in the fusion protein and linked from its C-terminus to the SH3 domains.

Despite the large variation in the length of the linkers tested, our preliminary studies based on pull-down experiments from Nef and Neffin transfected cell lysates, and affinity measurements with surface plasmon resonance did not reveal noticeable differences in the Nef-binding capacity of these Neffin variants, and all Neffin variants seemed to have greatly increased Nef binding potential compared to sdAb19 (data not shown). Therefore, we chose the seven-residue linker AAGGSGG construct for all further studies. To facilitate Neffin purification and detection, a C-terminal Myc-hexahistidine tail was added to this Neffin construct.

### Biochemical Properties of Neffins

**Figure 2:**
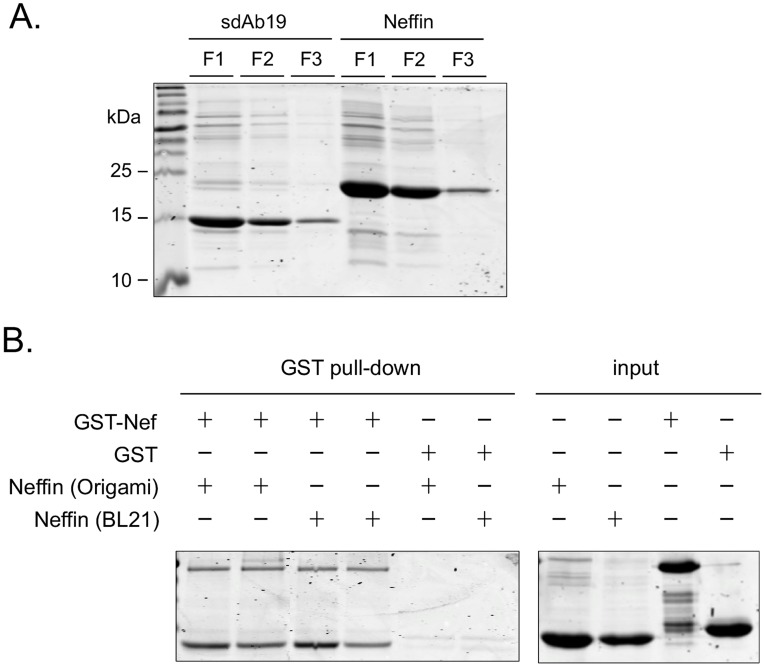
Bacterial expression of Neffin. (**A**) sdAb19 and Neffin were expressed in the cytoplasm of *E. coli* Origami cells in 50 ml flask cultures and captured to 0.2 ml of nickel-agarose resin. A Coomassie blue–stained gel containing 10 µl aliquots of the first three 0.5 ml fractions (F1–F3) of sdAb19 and Neffin eluated from the resin is shown. (**B**) Comparison of Nef-binding capacity of Neffin produced in BL21 or Origami cells. 10 µg (lanes 1 and 3) or 5 µg (lanes 2 and 4) of BL21- or Origami-derived Neffin were incubated with 10 µg GST-Nef or plain GST. Equal fraction of proteins captured to glutathione-resin well as input material were analyzed by SDS-PAGE and Coomassie blue–staining.

Due to small size and simple architecture of Neffin we hoped that its biochemical properties would be robust enough to enable large-scale production in soluble and functional form in the cytoplasm of *E. coli* without a need for targeting to periplasmic expression. When using a regular T7-derived bacterial vector large amounts of Neffin could be expressed in the cytoplasm of *E. coli* cells in regular flask cultures, and easily purified by standard nickel-resin affinity chromatography. With minimal optimization of the experimental conditions >18 mg/L of Neffin could be readily obtained (Fig. 2A). Of note, the amount of Neffin recovered from *E. coli* was consistently at least twice higher than the yields of the sdAb19 fragment expressed individually. No significant differences in the expression levels were observed when the BL21(DE3) *E. coli* cells were compared with thioredoxin reductase (trxB) and glutathione reductase (gor) deficient Origami(DE3) host cells (data not shown). Also, the proportion of functional protein was equally high in both cases, as similar amount of Neffins purified from BL21(DE3) or from Origami(DE3) cells could be re-captured to glutathione-S-sepharose beads coated with GST-Nef (Fig. 2B). Thus, we conclude that correct folding or disulphide bond formation did not limit high level cytoplasmic expression of functional Neffin proteins.

In summary, the VHH-SH3 double domain architecture seemed to be very well suited for bacterial expression, and the inclusion of the well-folding SH3 domain improved rather than compromised the favorable properties of the llama VHH fragment.

### Affinity for Nef

**Figure 3:**
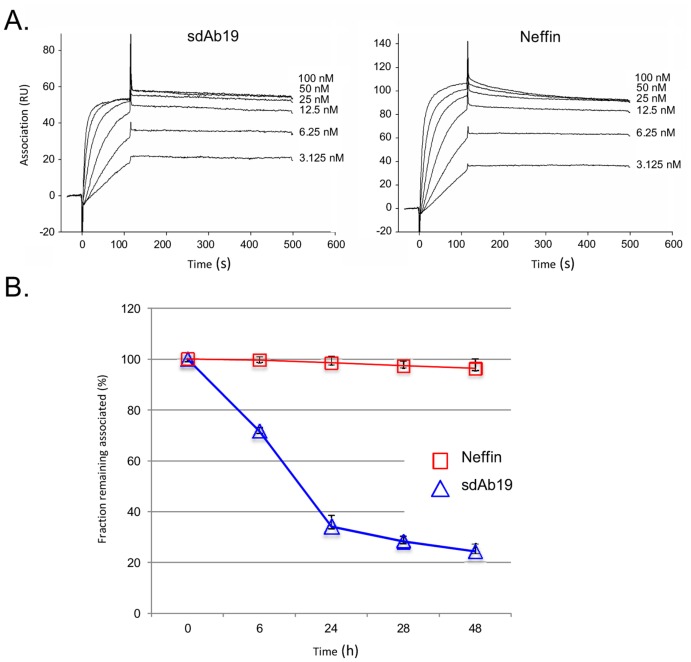
Estimation of binding affinity and kinetics for the Nef-Neffin interaction. (**A**) Biacore sensorgrams used for calculating the on-rates 1.41×10^6^ M^−1^s^−1^ and 1.51×10^6^ M^−1^s^−1^ for binding of sdAb19 and Neffin to Nef. The off-rates for both interaction were too slow to be reliably estimated by this method. (**B**)**.** Competitive ELISA for estimation of the off-rates of 2.17×10^−5^ s^−1^ and 8.12×10^−7^ s^−1^for binding of sdAb19 and Neffin to Nef.

To further examine the Nef-binding properties of Neffin, we immobilized GST-Nef onto a Biacore biosensor chip, and analyzed the association of different concentrations of sdAb19 or Neffins by surface plasmon resonance (Fig. 3A). When these curves were fitted to Langmuir 1∶1 model we found that both sdAb19 and Neffin bound to Nef with impressive on-rates (k_a_ of 1.41×10^6^ M^−1^s^−1^ and 1.51×10^6^ M^−1^s^−1^, respectively). The value recorded for sdAb19 is somewhat higher than published originally by Bouchet et al. [35]. We therefore carefully double-checked the concentration of the sdAb19 and Neffin-B6 preparations, generated independent new protein preparations, and repeated the measurements several times. The results were highly consistent leading us to conclude that sdAb19 binds to Nef with a remarkably rapid association rate, which is not significantly increased by fusion with SH3-B6.

It could be expected that the on-rate of binding cannot be increased by creating bivalent fusion proteins, and the potential gain of function would be provided by increased stability of binding. Indeed, evidence of slower dissociation of the Nef–Neffin complex as compared to the Nef–sdAb19 complex could be observed in exteneded Biacore runs with dissociation times much longer than those used in Fig. 3A (data not shown). However, the off-rate of sdAb19 was already slow (<10^−4^ s^−1^), and analysis of slow dissociation rates using Biacore is challenging [39], [40], [41]. To generate an experimental system suitable for examining the stability of the Nef–Neffin complex we set up an “ELISA-like” assay shown in Fig 3B.

In this assay microtiter plates were coated with the Nef protein, followed by incubation of sdAb19 or Neffin protein. After washing of the plates, an excess of soluble Nef protein was added to the wells to capture sdAb19/Neffin that dissociated from the immobilized Nef protein. At various times up to 48 hours the amount of sdAb19/Neffin that remained bound to the immobilized Nef was determined using a labeled antibody against the hexahistidine tag of sdAb19/Neffin. As shown in Fig. 3B the rate of dissociation from Nef was dramatically slower for Neffin compared to sdAb19. Based on these data dissociation rates of 2.17×10^−5^ s^−1^ and 8.12×10^−7^ s^−1^, respectively, were calculated.

When combining the association rates determined by Biacore with the dissociation rates determined by the off-rate ELISA, dissociation constants corresponding to the overall binding affinities could be calculated, K_d_ = 1.5×10^−11 ^M (15 pM, sdAb19) and 5.4×10^−13^ M (0.54 pM, Neffin). Thus, based on these analyses, we conclude that the VHH-SH3 fusion strategy resulted in synergistic bivalent binding of extreme affinity, and that Neffin bound to Nef 28-fold and more than 1000-fold better than its components sdAb19 and SH3-B6 (K_d_ = 12.3 nM; [38], see also Figure S2).

Since Biacore measurements can be sensitive to the surface density of the immobilized ligand it was important to examine if the tendency of GST to dimerize could have influenced our Biacore measurements based on GST-Nef immobilized onto the biosensor chips via an anti-GST antibody Therefore, we produced Nef as a fusion protein with the monomeric maltose binding protein (MBP), and compared binding of Neffin to GST-Nef and MBP-Nef covalently coupled directly onto Biacore chips. These experiments revealed identical sensorgrams (Figure S1), thus ruling out any significant contribution of GST dimerization to the recorded Neffin binding kinetics. We also tested MBP-Nef instead of GST-Nef in the off-rate ELISA, and again observed virtually identical results (data not shown).

### Neffin can Efficiently Bind and Inhibit Nef in Human Cells

The favorable biochemical properties of recombinant Neffin as well as the earlier studies on mammalian cell expression of SH3-B6 and sdAb19 [35], [36] suggested that the extreme Nef-binding capacity of Neffin might also be exploited in intracellular targeting and inhibition of Nef in human cells.

**Figure 4:**
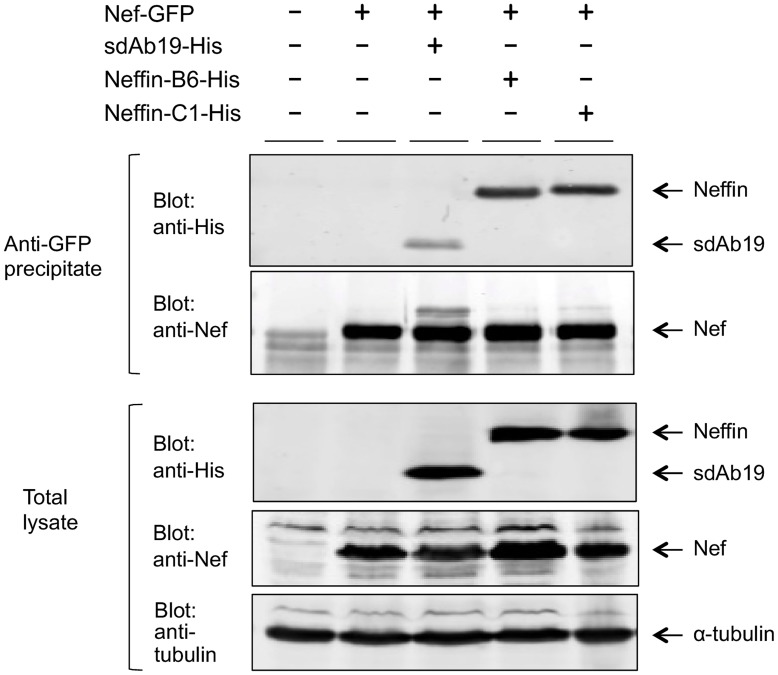
Efficient association of Neffin and Nef expressed in human cells. GFP-tagged Nef was co-expressed in 293 T cells together with sdAb19, Neffin-B6 or Neffin-C1, and the amount of sdAb19, Neffin-B6, or Neffin-C1 associated with anti-GFP immunocomplexes was determined by Western blotting (top panel). Even precipitation of GFP-Nef as well as equal expression of Nef, sdAb19, Neffin-B6, and Neffin-C1 in the lysates of the transfected cell cultures is shown as indicated. Blotting of the lysates with an antibody for the endogenous α-tubulin is used as a loading control. (bottom panel).

To this end, we transfected human 293 T cells with sdAb19 or Neffin expression constructs together with a vector expressing GFP-tagged Nef (Nef-GFP), and examined their ability to associate with Nef in these cells. In addition to the SH3-B6 containing Neffin (Neffin-B6 in Fig. 4), we included also another Neffin variant, namely Neffin-C1 (containing another Hck-derived Nef-targeted SH3 domain, SH3-C1, [22]) to this experiment. Forty-eight hours after transfection, the cells were lysed and Nef-GFP was immunoprecipitated, and the proteins in these precipitates were analyzed by SDS-PAGE and Western blotting. As shown in Fig. 4, when equal amounts of Nef-GFP together with sdAb19 or a Neffin were transfected into cells (see blotting of the total lysates), Neffins were very efficiently co-precipitated with Nef-GFP. While sdAb19 readily associated with Nef-GFP, the amount of co-precipitated sdAb19 was much weaker compared to Neffin-B6 and Neffin-C1. Thus, we concluded that the increased affinity of Neffin observed in vitro also translated in an enhanced association with Nef in human cells. Interestingly, for reasons that remain to be explored the gain in Neffin function provided by the SH3 moiety appeared to be even greater than found in using purified proteins in vitro.

**Figure 5:**
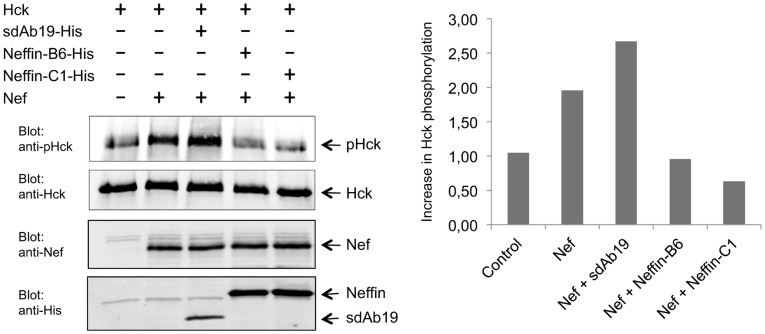
Potent inhibition of Nef-induced Hck activation by Neffin. An expression vector for the tyrosine kinase Hck was co-transfected to 293 T cell with an empty control vector (left lane) or with an expression vector for HIV-1 Nef (other lanes). In Nef-transfected cells a vector for sdAb19, Neffin-B6, or Neffin-C1 was included as indicated. Lysates of these cells were subjected to Hck-pulldown and the amount of activated (pHck; top panel) and total Hck (second panel) these precipitates determined by Western blotting. Corresponding amounts of Nef, sdAb19, Neffin-B6, and Neffin-C1 in the total lysates of the transfected cells was confirmed (third and bottom panels). A quantitation of relative Hck activation in the transfected cells normalized to the total amount of precipitated Hck is shown on the right, where the amount of autophosphorylated Hck in cells lacking Nef is set to one, and the phospho-Hck blotting signals from the other cells are graphed relative to this.

To study functional inhibition of Nef we chose to examine the capacity of sdAb19 and Neffins to suppress Nef-mediated enhancement of the catalytic activity of the Hck tyrosine kinase. This activation is caused by binding of Nef to the SH3 domain of Hck that is involved in keeping Hck in an enzymatically inactive conformation [42]. The activation of Hck was monitored using a phosphospecific antibody against the activation loop of Hck, which becomes autophosphorylated upon Hck activation. As shown in Fig. 5, Neffin-B6 and Neffin-C1 reduced the level of phospho-Hck to the baseline level seen in cells lacking Nef. Instead, in sdAb19-transfected cells Hck autokinase activity was induced as highly as in cells expressing only Nef with Hck. In addition to its lower capacity to associate with Nef in the transfected cells (Fig. 4), the failure of sdAb19 to suppress Hck activity may also be due to its mode of Nef binding, which does not lead to masking of the SH3 binding surface of Nef. Based on these data we concluded that Neffins could act as potent intracellular inhibitors of Nef.

## Discussion

In this study we describe a novel bivalent ligand binding protein constructed by fusion of a single-domain Ig heavy chain variable domain fragment with an optimized SH3 domain. The resulting fusion protein, named Neffin, remains very small in size (∼20 kDa) and could be readily produced in large amounts as a soluble and functional protein in *E. coli.* Yet, the binding of Neffin to its target protein, the HIV-1 pathogenicity factor Nef, showed very high affinity (estimated to be 0.54 pM) that can rarely be observed for natural or engineered antibodies, or the different types of recombinant ligand targeting proteins described so far.

The VHH-SH3 design therefore provides an interesting new approach for targeting of any protein of therapeutic or diagnostic importance. By immunizing camelids or via the use of synthetic gene libraries VHH fragments specific for a plethora other ligands can be generated. Based on earlier published work [28], [43], [44] as well as our own studies on unrelated VHH fragments (unpublished results), the robust and useful biophysical properties of sdAb19 are not a specific feature of this particular molecule, but instead appear to be shared by most VHH fragments of camelid origin. Likewise, ligand-tailored SH3 domains can be readily engineered for recognition of divergent target proteins of interest, including proteins that serve as ligands for natural SH3 domains as well as proteins that do not [21], [23]. To expedite finding of SH3 and VHH domains capable of co-operative bivalent binding rational screening approaches could be designed, for example by mixing one of the domains in excess in soluble form with the display library.

The published work on artificial, ligand-specific SH3 domains has relied on modification of SH3 domains derived from the Src-family tyrosine kinases [21], [22], [23], [45]. However, our recent unpublished studies on systematic testing of unrelated SH3 domains from divergent protein families have revealed even more suitable SH3 scaffolds for this purpose. Thus, due to its small size, efficient folding, and tolerance of extensive manipulation of its loop regions, the SH3 domain is an excellent scaffold for generating non-Ig-derived ligand-binding proteins for a variety of biotechnological and medical applications.

The concept of generating high binding affinity using multivalent recombinant proteins is not new, but has so far not been widely exploited. However, the simple design and robust properties of Neffin described here provide a strong case of the utility of this approach. The reasons for the rarity of existing applications of the multivalent binder concept are not clear, but are likely related at least in part to poor expression and solubility of many potentially useful protein binding domain combinations. By contrast, more than 18 mg/L of Neffin could be readily produced from regular flask cultures of *E. coli*, which is 10-times more than what is generally considered as a good yield for single-chain (scFv) or Fab antibody fragments. Moreover, this amount could be produced using cytoplasmic expression, thus circumventing the need for periplasmic targeting, thereby further simplifying and increasing the robustness of recombinant Neffin production. Because of these advantages the VHH-SH3 design has the potential to become a widely used approach to generate high-affinity recombinant ligand-binding proteins in a manner compatible with the practical and technical requirements of actual biotechnology applications.

The extreme binding affinity of the Neffin-Nef interaction can be traced to the strong Nef-binding capacities of its VHH and SH3 components individually. However, considering the remarkable ability for these two small domains to functionally co-operate, binding affinities superior to that of most antibodies could be achieved using VHH and SH3 components having more modest individual binding capacities compared to sdAb19 and SH3-B6. The affinity (*K_d_* 0.54 pM) measured for the Nef-Neffin interaction is indeed remarkable. This binding is much stronger than found in natural antibody-antigen interactions or in the majority of bioengineered interactions. Due to the challenge in accurately determining very slow dissociation rates, however, the overall affinity could be somewhat overestimated (but also underestimated). In this regard, it is possible that our ELISA-like off-rate assay based on surfaces immobilized with Nef might support more stable Neffin binding than what occurs in solution. We have also performed isothermal titration calorimetry (ITC) experiments to study the Nef-Neffin interaction in solution (data not shown). However, also this technique is poorly suited for determination of dissociation constants in the subnanomolar affinity range, and while the ITC studies did confirm the Nef-Neffin interaction to be of a very high affinity, no absolute value for the dissociation constant could be determined. In any case, it is clear that the VHH-SH3 design enables synergistic binding leading to small (∼20 kDa) proteins with binding capacity superior to that of typical antibodies.

Targeting the function of the viral pathogenicity factor Nef with the Neffin protein could have therapeutic applications in the management of HIV-1 infection. The ongoing progress in molecular medicine and research on gene therapy might allow efficient delivery of Neffin into the target cells of HIV-1. Blocking the intracellular function of Nef would be expected to have several beneficial effects, and could provide therapeutic synergy with the existing antiretroviral drugs. In this study we show evidence of inhibition of one of the intracellular functions of Nef, namely suppression of Nef-induced Hck tyrosine kinase activity. However, in another recent study we have extensively characterized the capacity of Neffin to inhibit a large panel of known cellular functions of Nef, and found that all these Nef-induced changes in the host cell behavior could be abrogated by Neffin co-expression (Bouchet et al., submitted).

Similar to the current development and use of antibodies and other affinity reagents for therapeutic purposes, the most obvious applications of ligand-specific VHH-SH3 proteins would be in blocking of extracellular targets involved in the pathogenesis of diseases like cancer and autoimmunity. However, the ease and versatility of generation and production of VHH-SH3 chimeras suggest that this approach could also be exploited to replace antibodies in a variety of other *in vitro* and *in vivo* practices in medicine as well as in industrial and biotechnology applications.

## Methods

### Bacterial Fusion Proteins

The cloning of GST-Nef_R71_ and MBP-Nef_R71_ has been described elsewhere [46]. GST-Nef, GST, MBP-Nef, and MBP were produced by introducing a corresponding plasmid vector into BL-21 strain of *E.coli* bacteria. The bacteria were grown to an optical density at 600 nm (OD_600_) of 0.6–0.8 (37°C, 250 rpm) followed by induction of protein expression with 1000 µM IPTG (isopropyl β-D-1-thiogalactopyranoside). Expression and purification of the GST proteins were carried out by standard methods as recommended by the supplier of the pGEX vectors and glutathione resin (Pharmacia). Expression and purification of the MBP proteins were carried out by standard methods as recommended by the supplier of the pMALC2× vectors (Novagen) and amylose agarose resin (Pharmacia).

In order to clone Neffin-B6 or sdAb19 into pET 12a bacterial expression vector, a DNA fragment encoding the Neffin-B6 or sdAb19 as well as the Myc and His-tag encoding sequences was amplified by PCR using the Fusion polymerase enzyme (Finnzymes) and primers containing the NdeI and BamHI site. This fragment was inserted into the corresponding sites in pET 12a vector, and used for expression of His-tagged fusion proteins without the OmpT leader sequence in the N-terminus of the fusion protein in Origami 2 *E.coli*. The bacteria were grown to an optical density at 600 nm (OD_600_) of 0.40 (37°C, 250 rpm) followed by induction of protein expression with 50 µM IPTG (isopropyl β-D-1-thiogalactopyranoside) for 21 hours (27°C, 220 rpm). Purification of the His-tagged fusion proteins were carried out by standard methods as recommended by the supplier of the Ni-NTA resin (Qiagen). Concentration measurements were performed using the BioRad (Lowry) method using bovine serum albumin as a standard.

### GST Pulldown

10 ug of purified GST-proteins (GST-R71 Nef or GST) were incubated with 10 ug or 5 ug of purified His-tagged sdAb19 or Neffin-B6 protein for 1 hour at +4°C in ELB pull down buffer (150 mM NaCl; 50 mM HEPES [*N*-2-hydroxyethylpiperazine-*N*′-2-ethanesulfonic acid], pH 7.4; 0.1% Igepal; 5 mM EDTA [ethylenediaminetetraacetic acid] and protease inhibitors (Roche)). Prewashed magnetic GST-sepharose beads (Promega, Madison,WI) were added to protein complexes and incubated for 1.5 h at +4°C. Pulldown samples were washed 3 times with the ELB buffer followed by SDS-PAGE and Coomassie protein analysis.

### Plasmids Used in Cell Transfection

Cloning of (WT) Nef_SF2_ into pEGFP-N1 vector (Nef.GFP) and sdAb19 into pcDNA3 vector has been described previously [35], [47]. Fusion proteins Neffin-B6 and Neffin-C1 were constructed by transferring an RRT-SH3 (B6 or C1) fragment from corresponding expression vectors into NotI site of sdAb19-pcDNA3 vector. Shortly, DNA fragment encoding the RRT-SH3 fragment was amplified by PCR using primers containing the NotI and EagI sites and nuleotides containing various linker peptides (see Fig. 1B), followed by insertion of this fragment into NotI site of sdAb19-pcDNA3 vector.

For Hck expression, an insert encoding wt human p61Hck was cloned into pEBB expression vector containing a C-terminal biotin acceptor domain (pp). The insert was PCR amplified from image clone 4855747 (GenBank: BC014435.1) plasmid template using primers containing the BglII and KpnI sites.The amplified insert was digested with indicated restriction enzymes and ligated into BamHI and KpnI restricted pEBB-pp plasmid.

### Cell Culture and Transfection

293 T human embryonic kidney cells were routinely cultured in a humidified 5% CO_2_ atmosphere at 37°C in Dulbecco modified Eagle medium (DMEM), supplemented with 10% (wt/vol) fetal calf serum (FCS), 1% L-glutamine and 1% penicillin-streptomycin. 293 T cells were transiently transfected using Fugene 6 transfection reagent according to the manufacturer’s instructions.

### Antibodies and Reagents

The following antibodies were used for experiments: Sheep polyclonal antibody to GST-Nef was a kind gift from Mark Harris (Leeds University, UK). Rabbit polyclonal antibodies against hexahistidine tag and Hck were purchased from Santa Cruz Biotechnology (Santa Cruz, CA). The rabbit polyclonal antibody to pHck (ab5203) was from Abcam (Fremont, CA) and rabbit polyclonal antibody to GFP (598) was from Nordic Biosite (Täby, Sweden). Mouse monoclonal anti-polyHistidine antibody (H1029, clone HIS-1), mouse monoclonal anti-α-Tubulin antibody (T6199) and goat polyclonal Anti-Mouse IgG HRP conjugate were from Sigma Aldrich (St. Louis, MO). The secondary IR-conjugated antibodies were from LI-COR Biosciences. Fugene 6 transfection reagent was purchased from Roche Diagnostics Corporation (Indianapolis, IN).

### Western Blotting

Tissue culture cells were lysed in KEB lysis buffer (137 mM NaCl, 50 mM Tris HCl [pH 8], 2 mM EDTA, 0.5% Igepal and protease inhibitors (Roche)) and subjected to immunoprecipitation using rabbit anti-GFP serum as described [47]. For determination of phosphorylation status of Hck, cells were lysed to *in vitro* kinase assay (IVKA) lysis buffer (150 mM NaCl; 50 mM HEPES, pH 7.4; 1% Triton X-100; 10% glycerol; 5 mM EDTA; 7.5 mM MgCl_2_ and protease and phosphatase inhibitors (Roche)).

Forty to sixty micrograms of total proteins were analyzed by 12–15% sodium dodecyl sulfate-polyacrylamide gel electrophoresis (SDS-PAGE) and blotted according to standard protocols. Protein detection was performed following incubation with appropriate first and IR-conjugated secondary antibodies followed by detection with Odyssey imager (LI-COR Biosciences) according to the manufacturer’s instructions.

### Immunoprecipitation and Pulldown

For detection of association between Nef-GFP and sdAb19 or Neffins, cell extracts from transfected 293 T cells were incubated with anti-GFP rabbit polyclonal antibody for 2 hours at +4°C. Immunocomplexes were coupled to protein A Sepharose beads for an additional 2 hour at +4°C and washed 3 times with the lysis buffer. For detection of pHck, cell extracts from Hck-pp-transfected 293 T cells were incubated with streptavidin beads (Invitrogen) for 2 hours at +4°C and washed 3 times with the lysis buffer. Immunoprecipitations and pulldown samples were subjected to SDS-PAGE and Western blotting analysis.

### Size Exclusion Chromatography

Analytical gel filtrations of recombinant Nef_NL4–3_, SH3-B6, sdAb19, and complexes thereof were performed using a multicomponent Waters 626 LC system (Waters, MA) equipped with a Superdex S75 (10/30) column (Amersham Biosciences). Typically, 100 µl of a 150 µg/ml protein solution was loaded onto the column that was equilibrated in 10 mM Tris/HCl (pH 9.0), 100 mM NaCl buffer prior to injection of the protein samples. Gel filtrations were run at a flow rate of 0.5 ml per minute in 10 mM Tris/HCl (pH 9.0), 100 mM NaCl onto the S75 column at 4°C. The optical density was monitored at a wavelength of 280 nm over the time course of the experiment. Gel filtration experiments were performed repeated times.

### Surface Plasmon Resonance

The affinities of sdAb19 and Neffin towards GST-Nef were analyzed by surface plasmon resonance in the Biacore 2000 biosensor instrumentation (GE Healthcare). First, flow cells of a CM5 biosensor chip were covalently coated with anti-GST antibody using the protocol provided in GST capture kit (GE Healthcare). In the beginning of each cycle, GST and GST-Nef at 10 µg/ml were captured on individual flow cells with a contact time of 4 min and flow rate of 5 µl/min. The captured GST and GST-Nef repeatedly gave an increase of 800–1000 resonance units (RU) in baseline signal. The binding of various concentrations of sdAb19 and Neffin ranging between 3.125–100 nM were analyzed with a 2 min contact time and a subsequent 15 min dissociation phase at a flow rate of 50 µl/min. At the end of each cycle the surface was regenerated with 1 min pulse of 10 mM Glycine (pH 1.7). The analytes were diluted in PBS running buffer which was supplemented with 0.0005% p20. For analyte concentrations of 6.25 nM the dissociation phase was also separately recorded for a period of 2 h.

The data were evaluated by subtracting sensorgrams obtained from GST-coated flow cells from those obtained with GST-Nef. The subtracted sensorgrams were fitted to a Langmuir model assuming 1∶1 binding using BiaEvaluation Software 3.1 (GE Healthcare).

### Off-rate ELISA

Off-rate ELISA was performed in 96-well Maxisorp™ microtiter plates (Nunc, Langenselbold, Germany) coated over night at 4°C with 100 µl of MBP-Nef antigen (5 µg/ml in PBS). The wells were washed 3×with PBS-0.05% Tween20 and blocked with 5% skimmed milk powder in PBS for 2 h at RT. Appropriate dilutions of soluble binders were prepared in 2× YT and incubated with the coated antigen for 1 h at RT followed by washes 5×with PBS-0.05% Tween20 to remove unbound binders. At this starting point (0 h), 100% of the binders were in complex with the antigen and no free binder existed. Dissociation kinetics of the binder-antigen complex was then monitored as a function of time using MBP-Nef as a specific capture antigen to inhibit reassociation of the dissociated binders, whereas MBP served as an irrelevant control antigen. Specifically, three parallel wells were incubated in the presence of an excess of MBP-Nef or MBP (100 µl of 300 nM antigen in PBS) for diverse periods of time (0–48 h) followed by washes 5×with PBS-0.05% Tween20 to remove dissociated binders. Functionality of the capture and control antigens was controlled by coincubation of the binders with the antigens prior to exposure to MBP-Nef-coated wells. The detection was performed with mouse monoclonal anti-His-HRP antibody, which recognizes the C-terminal His-tag of the binders, and TMB (3,3′ 5,5′-tetramethylbenzidine) substrate. The staining reaction was stopped with 1 M sulfuric acid and absorbance measured at 450 nm using Multiskan Ascent ELISA-reader (Thermo Fisher Scientific). The mean absorbance values of triplicate samples were normalized relative to the control. ( = 1).

Under first-order conditions, the kinetic dissociation constant k_d_ is directly related to the half-life of the bimolecular complex (t_1/2_) in irreversible dissociation conditions through the equation: t_1/2_ = −ln 0.5/k_d_, establishing an intuitive and direct relation between k_d_ and life-time of the complex [48]. Consequently, normalized mean absorbances, reflecting the proportion of bound binder at a given point of time, were used for calculation of k_d_ through the following equation: k_d_ = −ln (Normalized absorbance)/t.

## Supporting Information

Figure S1 **Surface plasmon resonance analysis to compare binding kinetics of Neffin to immobilized GST-Nef and MBP-Nef proteins.** Nef fusion proteins were covalently coupled directly onto CM5 biosensor chips (GE Healthare) and 100 nM of Neffin was injected as an analyte. The maximal binding signals are indicative of the amount of functional MBP- Nef and GST-Nef proteins immobilized, whereas the matching shapes of the sensorgrams indicate very similar binding kinetics in both cases. Similar to the data in Fig. 3A the off-rates were too slow to be meaningfully determined, while the on-rates matching well with data in Fig. 3A were obtained.
(TIF)

Figure S2 **Surface plasmon resonance analysis of the individual Nef binding capacity of SH3-B6.** SH3-B6 was expressed as a His-tagged MBP-fusion protein and immobilized onto an NTA chip. Different concentrations of GST-Nef were then injected as indicated. The association and dissociation rate constants were obtained by fitting the obtained sensorgrams to a Langmuir global fit model assuming 1∶1 binding.
(TIF)
