# Supplementary figures and images for: High-Affinity Target Binding Engineered via Fusion of a Single-Domain Antibody Fragment with a Ligand-Tailored SH3 Domain
Source: PLoS One. 2012 Jul 5;7(7):e40331. doi: 10.1371/journal.pone.0040331 (PMC3390362; doi:10.1371/journal.pone.0040331)

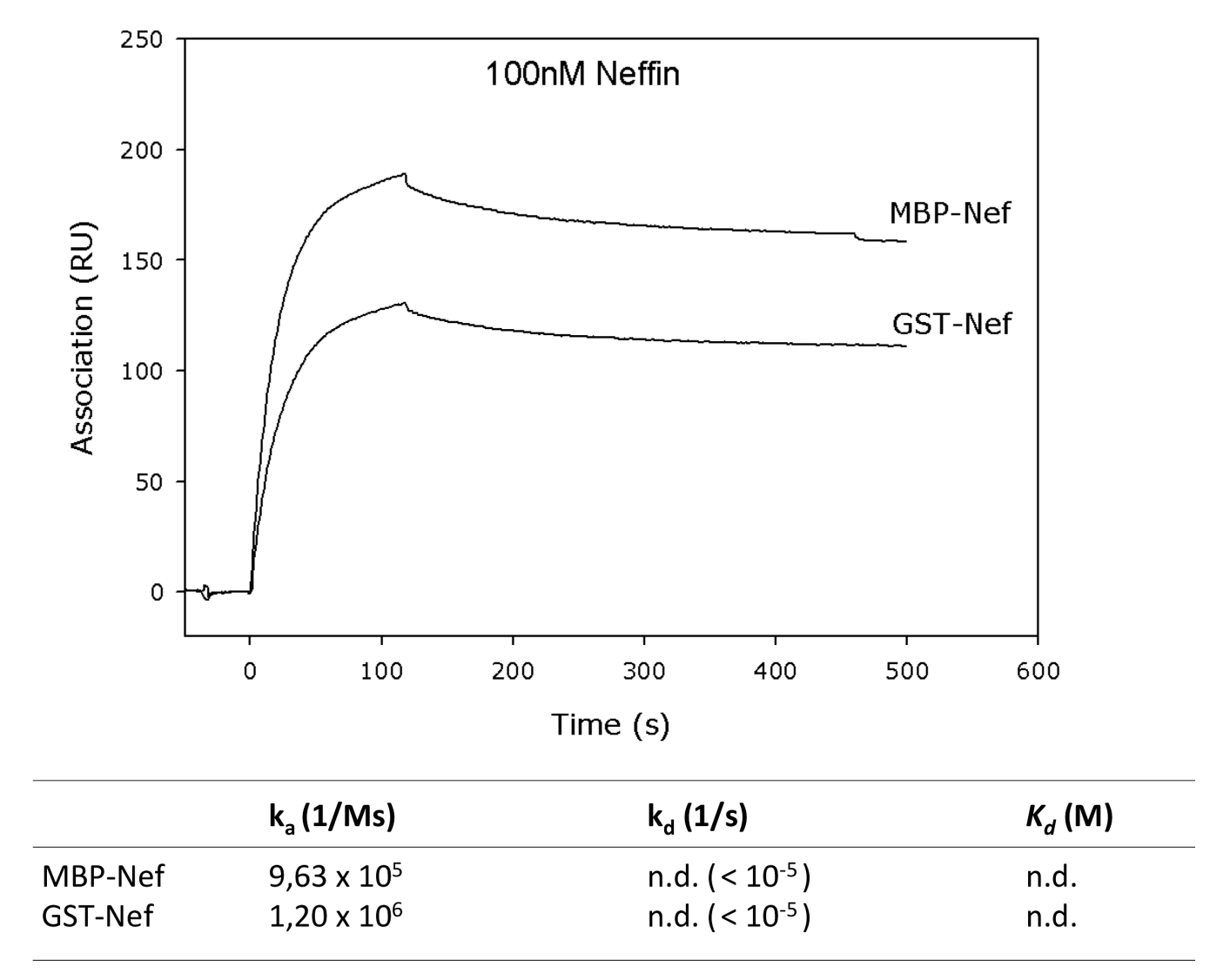

Supplement: Figure S1 — Surface plasmon resonance analysis to compare binding kinetics of Neffin to immobilized GST-Nef and MBP-Nef proteins. Nef fusion proteins were covalently coupled directly onto CM5 biosensor chips (GE Healthare) and 100 nM of Neffin was injected as an analyte. The maximal binding signals are indicative of the amount of functional MBP- Nef and GST-Nef proteins immobilized, whereas the matching shapes of the sensorgrams indicate very similar binding kinetics in both cases. Similar to the data in Fig. 3A the off-rates were too slow to be meaningfully determined, while the on-rates matching well with data in Fig. 3A were obtained. (TIF) [file pone.0040331.s001.tif]

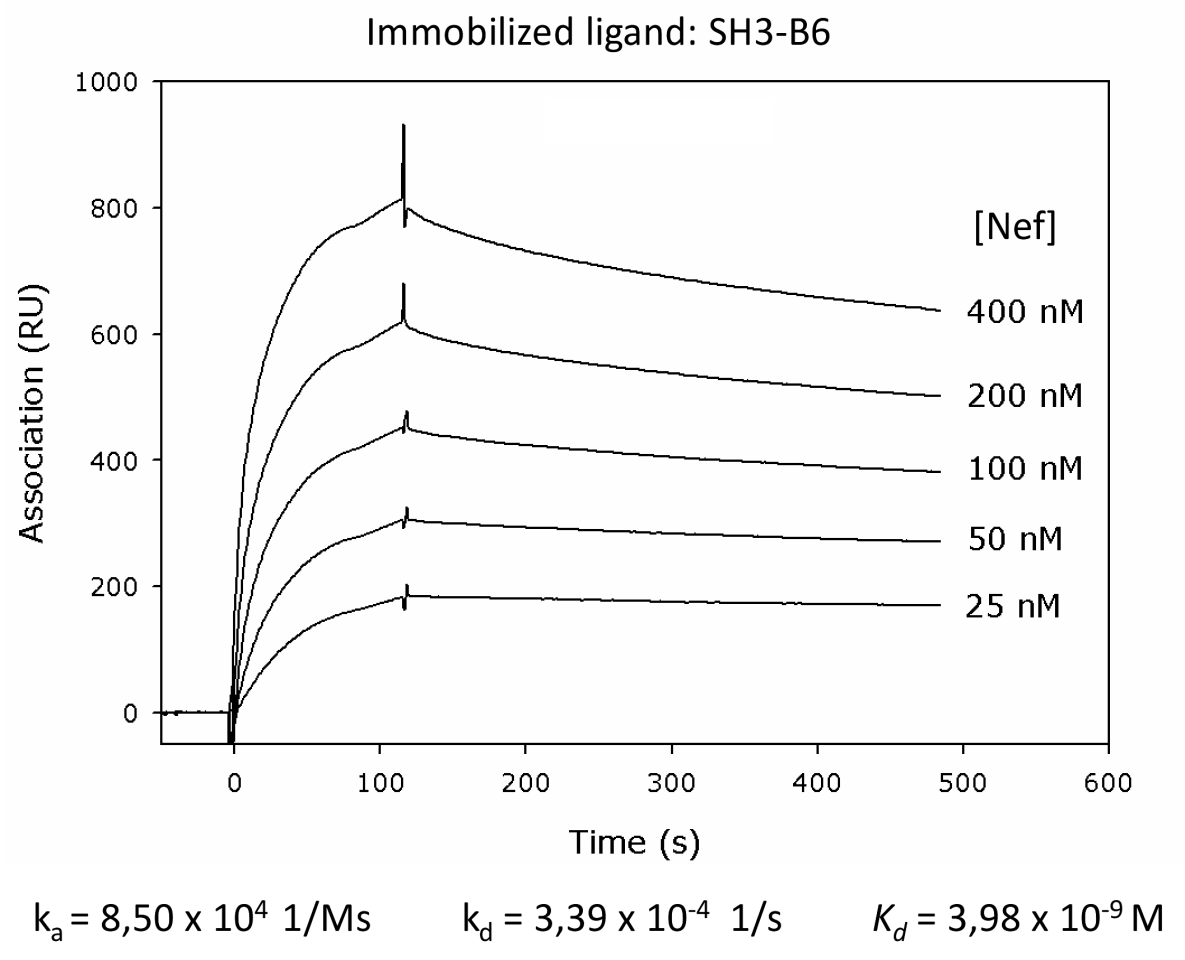

Supplement: Figure S2 — Surface plasmon resonance analysis of the individual Nef binding capacity of SH3-B6. SH3-B6 was expressed as a His-tagged MBP-fusion protein and immobilized onto an NTA chip. Different concentrations of GST-Nef were then injected as indicated. The association and dissociation rate constants were obtained by fitting the obtained sensorgrams to a Langmuir global fit model assuming 1∶1 binding. (TIF) [file pone.0040331.s002.tif]
